# Egg Viability, Mating Frequency and Male Mating Ability Evolve in Populations of *Drosophila melanogaster* Selected for Resistance to Cold Shock

**DOI:** 10.1371/journal.pone.0129992

**Published:** 2015-06-11

**Authors:** Karan Singh, Ekta Kochar, N. G. Prasad

**Affiliations:** Indian Institute of Science Education and Research Mohali, Knowledge City, Sector 81, SAS Nagar, PO Manauli, Punjab 140306, India; University of Natural Resources and Life Sciences, Vienna, AUSTRIA

## Abstract

**Background:**

Ability to resist temperature shock is an important component of fitness of insects and other ectotherms. Increased resistance to temperature shock is known to affect life-history traits. Temperature shock is also known to affect reproductive traits such as mating ability and viability of gametes. Therefore selection for increased temperature shock resistance can affect the evolution of reproductive traits.

**Methods:**

We selected replicate populations of *Drosophila melanogaster* for resistance to cold shock. We then investigated the evolution of reproductive behavior along with other components of fitness- larval survivorship, adult mortality, fecundity, egg viability in these populations.

**Results:**

We found that larval survivorship, adult mortality and fecundity post cold shock were not significantly different between selected and control populations. However, compared to the control populations, the selected populations laid significantly higher percentage of fertile eggs (egg viability) 24 hours post cold shock. The selected populations had higher mating frequency both with and without cold shock. After being subjected to cold shock, males from the selected populations successfully mated with significantly more non-virgin females and sired significantly more progeny compared to control males.

**Conclusions:**

A number of studies have reported the evolution of survivorship in response to selection for temperature shock resistance. Our results clearly indicate that adaptation to cold shock can involve changes in components of reproductive fitness. Our results have important implications for our understanding of how reproductive behavior can evolve in response to thermal stress.

## Introduction

Environmental stress such as extreme temperatures, desiccation, crowding can have major consequences for fitness of organisms. In holometabolous insects like *D*. *melanogaster*, different life stages (which live in different environments), can experience different types of stresses. The ability to resist environmental stress is an important component of fitness. Using *Drosophila*, a large number of studies have investigated the evolution of resistance to various environmental stresses such as larval crowding [1], increased urea and ammonia content in larval food [2], adult desiccation, starvation [3, 4] and extreme temperatures [5–9].

Temperature is one of the most important ecological factors that affect the fitness of an organism [10]. This is especially true of insects which are ectothermic [11, 12]. Effects of high temperature shock on fitness are well studied [13–17]. High temperature shock (i.e., heat shock) can affect longevity, survivorship, fecundity and male fertility [16–24]. Just like heat shock, sudden exposure to sub-zero temperature (i.e., cold shock) can also potentially affect ectotherms.

Cold shock decreases the survivorship of adults, eggs and pupae and reduces fecundity [5–9, 23–28]. Consequently, insects have evolved a number of mechanisms to deal with cold stress [29]. Some studies have addressed the evolution of resistance to cold shock See [30] for detail account. A study across 95 species of *Drosophila* indicated that evolutionary responses in cold resistance are likely to be slow because of considerable phylogenetic inertia [31]. Other studies have found clinal variation in traits related to cold tolerance in populations of *Drosophila* [32–35]. Results from laboratory studies suggest that populations selected for resistance to cold shock evolve increased survivorship post cold shock [5, 6, 9]. Other studies show that selection for faster recovery from chill induced coma in *D*. *melanogaster* leads to a correlated increase in adult survivorship and fecundity post cold stress [8]. However, longevity of the selected populations was significantly lower than that of the controls (when measured in the absence of cold shock). Increased tolerance to cold shock has not been found to be correlated with resistance to other stresses such as heat shock [8], starvation and desiccation [9].

Apart from affecting life-history traits such as fecundity and longevity, cold shock can also affect reproduction by decreasing sperm stock [36] as well as mating ability of males [14, 36]. In *D*. *melanogaster*, females are de-seminated by cold shock treatment [37]. Previous study documented that cold shock kills sperm in the storage organs of female *D*. *melanogaster* [38]. The dead sperm are then ejected by the females. Their future fecundity is, therefore, dependent upon re-insemination. Cold shock also kills the mature sperm stored in the seminal vesicles of the male *D*. *melanogaster* [38]. The males do not show any motile sperm for a period of 24 hours after treatment and fail to transfer any sperm to females during this period. Thus, the future fitness of cold-shocked males depends upon their ability to produce fertile sperm and successfully mate with females post cold shock [38]. Thus, it is very clear that cold shock can severely affect fitness of *D*. *melanogaster* through its effects on reproductive traits such as sperm production, storage and mating. Therefore, it is very likely that adaptation to cold shock will involve changes in reproductive physiology. Some studies have addressed the evolution of life-history traits in response to selection for resistance to cold shock (see references above). However, to the best of our knowledge, no study has so far addressed the evolution of reproductive behavior in response to selection for resistance to cold shock.

In the present experiment, our major goal was to address the evolution of components of reproductive fitness such as mating frequency and mating ability in response to cold shock. We subjected adult flies to a non-lethal cold shock (-5°C for one hour) which reduced egg viability (defined as the proportion of the eggs that hatch) by nearly 40% but had relatively little effect on adult mortality (about 3–9%). Thus, in our study, selection was primarily on egg viability. The selection protocol we used was similar to that of several previous studies that have addressed the evolution of cold shock resistance [5–7, 9]. The major focus of these previous studies has been the evolution of adult mortality and other life-history traits. In our study, we focused on the evolution of reproductive traits.

The study consisted of 10 populations of *D*. *melanogaster* (5 selected populations and 5 control populations) and was conducted over 30 generations of selection. We specifically addressed the following questions;

(a) Does egg viability post cold shock evolve as the primary response to selection?
(b) Do fecundity, larval mortality and adult mortality post cold shock evolve as a correlated response to selection?

As noted before, cold shock can kill stored sperm in males and females [37, 38]. Hence, we also tested whether -

(c) The ability of the females to protect the sperm from cold shock has evolved and
(d) The ability of the males to mate successfully and sire progeny post cold shock has evolved.

We found that larval survivorship, adult mortality and fecundity post cold shock did not evolve. However, egg viability and components of reproductive behavior had evolved in the selected populations.

## Materials and Methods

### Base line population

In 2010 nineteen isofemale lines of *D*. *melanogaster* were established using wild inseminated females collected from Blue Ridge, Georgia, USA and maintained in the laboratory of Daniel Promislow at University of Georgia, USA. These lines were kindly provided to us in 2010, after which they were maintained in our laboratory for 6 generations. In 2011, we combined 100 males and females from each of the 19 isofemale lines to create a single large population of *D*. *melanogaster* called Blue Ridge Base line (BRB). The BRB population was maintained for ten generations under standard laboratory conditions (see below) after which it was split into 5 replicate populations called BRB 1–5 (see the flow diagram in S1 Fig).

The five BRB populations are maintained on a 14 day discrete generation cycle at 25°C temperature, 50–60% RH, 12:12 hours light-dark cycle on standard banana-yeast-jaggery food [39]. Eggs are collected from adult flies and dispensed into glass vials (25mm diameter **×** 90mm height) containing 6ml of banana-yeast-jaggery food at a density of about 70 eggs per vial and incubated at standard laboratory conditions (see above). Forty such vials are set up per population. On 12th day post egg collection (by which time almost all the adults eclose), the adults are transferred to Plexiglas cages (25cm length **×** 20cm width **×** 15cm height) provided with a Petri plate containing standard banana-yeast-jaggery food supplemented with live yeast paste (ad-lib). Each cage contains approximately 2800 adults. On the 14th day post egg collection, fresh food plates are provided in the cages for 18 hours and the eggs are collected from these plates to start the next generation. The BRB 1–5 populations were maintained under the above mentioned conditions for 35 generations before starting the present study.

### Derivation and maintenance of selected and control populations

After 35 generations of standard laboratory maintenance, we derived one selected (FSB) and one control population (FCB) from each of the five BRB populations (S1 Fig). Thus the experiment consisted of ten populations in all- five selected populations (FSB 1–5) and five control populations (FCB 1–5). The selected and control populations with the same numerical subscript are more closely related to each other than to other selected or control populations. For example, FSB 1 and FCB 1 are derived from BRB 1 and are more closely related to each other (by ancestry) than to FSB 2. The pair of selected and control populations bearing the same numerical subscript are thus treated as statistical blocks. For example, FSB 1 and FCB 1 constitute Block 1 and so on. Thus, the experiment consisted of five Blocks. During maintenance and experiments, populations from the same block were always handled together. For example, FSB 1 and FCB 1 were always handled together during maintenance and experiments.

The selected populations (FSB 1–5) are maintained on a 13 day discrete generation cycle (S2 Fig). Eggs are collected from adults and dispensed into vials (25mm diameter **×** 90mm height) containing about 6ml of standard banana-yeast-jaggery food at a density of about 100 per vial. Twenty such vials are set up per population. The vials are incubated at 25°C temperature, 50–60% RH and 12:12 light:dark cycle. The flies start eclosing by the 9th day post egg collection with peak eclosion happening on the 10th day post egg collection. On day 12 post egg collection (by which time all the adults eclose and are about 2 days old post eclosion), the adults from each of the 20 vials are transferred into 20 dry, empty vials (about 60 to 70 flies per vial). The cotton plug is pushed in such that the flies are confined to a small area at the bottom one third of the vial. These vials are then placed into salt-water-ice slurry maintained at -5°C and held there for one hour. Care is taken to see that the part of the vial containing the flies is completely immersed in the slurry. After one hour, the flies are transferred into a Plexiglas cage containing a Petri plate of banana-yeast-jaggery food and maintained at 25°C temperature.

Twenty four hours after the cold shock treatment (i.e., 13th day post egg collection), the flies are provided with a fresh food plate and are allowed to oviposit for 18 hours. Hence, during normal maintenance regime, eggs are collected to start the next generation in a 18-hour window lasting between 24–42 hours post cold shock. These eggs are then dispensed into food vials to start the next generation. It is important to note that the cold shock treatment that we use causes low adult mortality (3 to 9%). However, the viability of the eggs laid 24 hours post cold shock is reduced by about 30–40%. Therefore, in our study, selection is primarily on egg viability 24 hours post cold shock. For the FSB populations, we collect eggs at a density of about 100 per vial such that the numbers of emerging larvae and adults is approximately, 60–70 per vial. Thus there are about 1200 to 1400 adults per generation per population.

The control populations (FCB 1–5) are maintained under exactly the same conditions as the selected (FSB 1–5) populations (S2 Fig) but for the following changes

1. On 12th day post egg collection, the flies from the FCB population are transferred into empty vials and are held in a water bath maintained at 25°C for one hour.
2. The eggs are collected from the adults and dispensed into food vials at a density of 60–70 per vial.

### Standardization of flies

To account for differences between selected and control populations due to non-genetic parental effects [40], all the populations (FSB1-5 and FCB 1–5) were reared under laboratory conditions as described below for one generation. During this generation, the FSB populations were not subjected to selection. We call this process ‘standardization’ and the flies ‘standardized flies’. For standardization, eggs are collected from each of the FCB and FSB stock populations. Eggs from a given population are distributed into vials containing standard banana-yeast-jaggery food at a density of about 70 eggs per vial. 20 such vials are set up per population. The vials are incubated at standard laboratory conditions (see above). On 12th day post egg collection, the adults from a given population are transferred into a Plexiglas cage provided with banana-yeast-jaggery food. These flies are called the standardized flies. On 13th day post egg collection, a fresh food plate is provided and the standardized flies are allowed to oviposit for 6 hours. Using moist camel-hair paint brushes, eggs are collected from these food plates and dispensed into vials containing 6ml of standard banana-yeast-jaggery food at an exact density of 70 eggs per vial. Adults emerging from these vials (i.e., the progeny of the standardized flies) are used for further assays. Most of the experiments reported in the present study were conducted after 19 to 30 generations of selection.

### Experimental protocol

#### Cold shock treatment

Flies are transferred to clean dry glass vials (25 mm diameter **×** 90 mm height) on 12th day post egg collection (2–3 days post eclosion) at a density of 50 individuals per vial (in mixed sex groups or single sex groups as per the requirement of the experiment) under light carbon dioxide anesthesia. The cotton plug is pushed deep into the vial such that the flies are restricted to the bottom one third of the vial. The flies are allowed to recover from anesthesia for half an hour. The vials are then placed in water-ice-salt slurry maintained at -5°C and allowed to stand for one hour. The flies are then transferred to Plexiglas cages (14cm length **×** 16cm width **×** 13cm height) at a density of 100 pairs per cage in case of mixed sex treatment and 100 flies per cage in case of single sex treatment. The cage is provided with a Petri plate containing standard banana-jaggery food and is maintained under standard laboratory conditions (see above).

The flies from the control treatment are handled identically except that the dry glass vials containing the flies are transferred to a water bath maintained at 25°C for one hour. The males and females are held together at a density of 100 pairs per cage.

#### Experiment 1: Have egg viability, fecundity and mating frequency post cold shock evolved?

As noted before, cold shock that we use reduces egg viability by about 40% but causes about 3–9% mortality in the adults. Therefore, in our experiment, egg viability is primarily under selection. Hence, we first assayed the direct response to selection in terms of egg viability 24 hours post cold shock. Using the same experimental set up, we also collected data about fecundity, adult mortality and mating frequency of the selected and control populations. See details of the experimental design in the pictorial representation (S3 Fig).

We specifically asked the following questions- are egg viability and fecundity affected by (a) treatment i.e., Cold shock vs. Neither-cold shock (b) selection history (c) time gap between cold shock and egg viability measurement and (d) access to healthy vs. cold shocked mates.

After 19 generations of selection, eggs were collected from standardized flies as described above. Thirty nine vials were set up for each of the ten populations. For each of the FSB and FCB populations, on 12th day post egg collection (by which time all the flies had eclosed and mated) the vials were randomly assigned to one of the following three treatments.

1. Both-shocked: both males and females from a given population were subjected to cold shock (as described before) and were then transferred to a Plexiglas cage at a density of 100 pairs per cage.
2. Female-shocked: only females (non-virgin) from a given population were subjected to cold shock (as described before). Following the cold shock, they were transferred into Plexiglas cages at a density of 100 females per cage. We then added 50 non-shocked males from the corresponding BRB base population to the cage. For example, a cage containing 100 FSB 1 cold shocked females received 50 non-shocked BRB 1 males. Thus the shocked females had access to healthy males for one day post cold shock. We deviated from 1:1 sex ratio under the assumption that cold shock imposes stress and/or physiological harm to the females [9]. Given this, we wanted to minimize the confounding factor of physical mate harm imposed by males (e.g., unsuccessful courting) while making sure that there are enough males to inseminate all the females.
3. Neither-shocked: Neither the males nor the females were subjected to cold shock. Instead, the males and females from a given population were held in a water bath maintained at 25°C for one hour. They were then transferred to Plexiglas cages at a density of 100 pairs per cage.

We assayed fecundity and egg viability at two points—(a) 0 hours post cold shock and (b) 24 hours post cold shock. We chose these two time points because (a) measures of fecundity and egg viability at 0 hours post cold shock represents the immediate effect of cold shock and (b) 24 hours post cold shock is the time that eggs are collected from the flies to start the next generation in their normal maintenance regime and is hence directly relevant to the fitness of the flies. At the beginning of each time period, dead flies (if any) were aspirated out of the cage and counted. A fresh food plate was provided in the cage for the females to lay eggs for 6 hours. A sample of 200 eggs from the food plate were then moved to a Petri plate containing 1.2% agar and incubated at 25°C for 30 hours, after which the numbers of hatched eggs were counted to obtain an estimate of the viability of the eggs. The rest of the eggs in the food plate were counted to measure total fecundity. This value was divided by the number of females that were alive at the start of the 6-hour oviposition window to obtain fecundity per female. To assess the effect of cold shock on adult mortality, we used the adult mortality values from the Neither-shocked and both-shocked treatments.

In blocks 1, 2, 4 and 5 we set up three cages per Selection **×** Treatment combination. However, in block 3, there were two cages per Selection **×** Treatment combination. Fecundity and egg viability were assayed from each cage as described above. We used the fecundity per female and egg viability values from each cage as the units of analysis.

We also quantified the total number of matings in the Both-shocked and Neither-shocked treatments. Once the flies were transferred into a cage, we observed them every half an hour and noted the total number of mating pairs. These observations were carried out until 36 hours post cold shock. In the normal maintenance regime, eggs are collected from the flies to start the next generation in an 18 hour window between 24–42 hours post cold shock. In *Drosophila*, post mating, a majority of the females finish processing the sperm (transport of sperm to seminal receptacle, ejection of sperm from bursa etc.) in about 5 hours [41]. Hence, matings that happen until about 36 hours post cold shock can, in principle, result in progeny. Hence we chose to observe mating until 36 hours post cold shock. We then summed the number of mating pairs across all the observations for a given cage to obtain an estimate of the total number of matings. The total number of mating pairs per cage was used as the unit of analysis.

#### Experiment 2: Has the larval survivorship post cold shock evolved in the selected populations?

We wanted to test whether our selected populations have evolved better larval survivorship post cold shock. After 30 generations of selection, we assessed the effect of cold shock on survivorship of larvae from the selected and control populations. Eggs were collected from standardized flies as described before. Ten such vials were set up per population. On 12th day post egg collection (by which time all adults had eclosed), the vials from each population were randomly assigned to one of the two treatments- (a) subjected to cold shock or (b) subjected to control (25°C) treatment. The flies were transferred to Plexiglas cages at a density of approximately 150 pairs per cage. Twenty four hours later, eggs were collected from the cages by providing them with fresh food plates for a four hour window. The food plates containing the eggs were then incubated at 25°C. Twenty four hours later, when the eggs had hatched and the first instar larvae had emerged, using a fine, moist paint brush, we transferred the first instar larvae into fresh food vials at a density of 30 larvae per vial. Ten such vials were set up per population. The vials were then incubated under standard laboratory conditions for 15 days and all the emerging adults were counted. Thus each vial yielded a value of larva to adult viability. These vial values were used as the units of analysis.

#### Experiment 3: Are females from the selected populations better at protecting eggs/stored sperm from cold shock?

Previous studies suggest that cold shock kills the sperm in female storage organs. Thus insemination post cold shock is necessary for the females to lay fertile eggs. However, it is possible that females from the selected populations are better able to protect sperm from cold shock. To assess this possibility, we carried out an experiment after 30 generations of selection. Eggs were collected from standardized flies as described before. Twenty four such vials were set up per population. On 12th day post egg collection, the vials from each population were randomly assigned to one of the two treatments- (a) subjected to cold shock or (b) subjected to control (25°C) treatment. For both the treatments, females were separated from the males (under light carbon dioxide anesthesia) and were transferred into clean, dry glass vials. The males were discarded. The females were then subjected to their respective treatments (as described before) and transferred to Plexiglas cages at a density of 100 females per cage. Thus the females (from the cold shock treatment and the control treatment) had no further access to males. Twenty four hours later, we assayed fecundity and egg viability of the females from the two treatments as described before. We set up three cages per Selection **×** Block **×** Treatment combination. Fecundity and egg viability values from each cage were used as the units of analysis.

#### Experiment 4: Has the ability to sire progeny post cold shock evolved in males of our selected populations?

We wanted to test if the ability to sire progeny post cold shock had evolved in our selected populations. After 19 generations of selection, we assessed the ability of cold shocked males from the FSB and FCB populations to mate non-shocked, non-virgin females and sire progeny. Eggs were collected from standardized flies as described before. Ten such vials were established per population. On 12th day post egg collection, males from each of the vials were separated from the females under light carbon dioxide anesthesia and subjected to cold shock as described before. One hundred males were then transferred into a Plexiglas cage that contained 100 non-shocked, non-virgin females from an unrelated base stock called LHst which contains a recessive scarlet eye color marker [42].

The LHst females were generated by collecting eggs from the LHst base population at a density of 70 eggs per vial and incubating the vials containing standard cornmeal-molasses-yeast food at standard laboratory conditions [43]. The flies started eclosing by 9th day post egg collection with peak eclosion on 10th day post egg collection. The LHst males and females continued to interact with each other in the same vials for a further two days (by which time all females are mated). On 12th day post egg collection, females were isolated using light carbon dioxide anesthesia and transferred into Plexiglas cages just before the males from the FSB and FCB populations were introduced into the cages.

The FSB/FCB males were allowed to interact with the LHst females for 24 hours post shock after which time, 50 LHst females from each cage were randomly sampled using carbon dioxide anesthesia and transferred individually into test tubes (12mm diameter × 75mm length) containing standard banana-yeast-jaggery food. The females were allowed to oviposit for 18 hours after which the females were discarded and the test tubes were incubated under standard laboratory conditions. Thirteen days later, progeny emerging from each of these tubes were counted and their eye color was noted. The males from the FSB/FCB populations have a dominant red eye color while the females from the LHst populations have a recessive scarlet eye color. Hence the progeny from the mating of LHst females with FSB/FCB males will have red eye color while the progeny from the previous matings of LHst females (with LHst males) will have scarlet eye color.

We noted the number of females that produced progeny with red eye color (since this indicates successful mating with FSB/FCB males at least once). We also calculated the proportion of red eyed progeny within the total progeny pool produced by the LHst females. Thus each cage yielded one value for proportion of LHst females mating with males of interest (i.e., FSB or FCB males) and one value for proportion of progeny sired by males of interest. These values were used as the units of analysis.

### Statistical analysis

Since selected and control populations bearing the same numerical subscript were derived from the same BRB population, they are more closely related to each other than they are to any other population. For example, FSB 1 is more closely related to FCB 1 (since they both were derived from BRB 1) than to FSB 2. Hence they are treated as statistical blocks in all the analyses.

For experiment 1, fecundity and egg viability were analyzed using a four factor, mixed model Analysis of Variance treating Selection regime (FCB vs. FSB), Treatment (Neither-shocked, Both-shocked and Female-shocked) and Period (0 hours vs. 24 hours post cold shock) as fixed factors with Blocks (1–5) as random effect. Adult mortality data were analyzed using a four factor, mixed model Analysis of Variance treating Selection regime (FCB vs. FSB), Treatment (Neither-shocked and Both-shocked) and Sex (male and female) as fixed factors with Blocks (1–5) as random effect. Mating frequency data from Experiment 1 along with data from experiment 2 and 3 were analyzed using a three factor mixed model Analysis of Variance treating Selection regime and Treatment as fixed factors crossed with random Blocks. Data from experiment 4 (proportion of non-virgin females mated by FCB vs. FSB males after cold shock; proportion of progeny sired by FCB vs. FSB males after cold shock) were subjected to paired t tests (two tailed). Multiple comparisons were done using Tukey's HSD.

## Results

### Experiment 1: Egg viability and mating frequency post cold shock have evolved in the selected populations. However, fecundity and adult mortality post cold shock have not evolved

**Fig 1:**
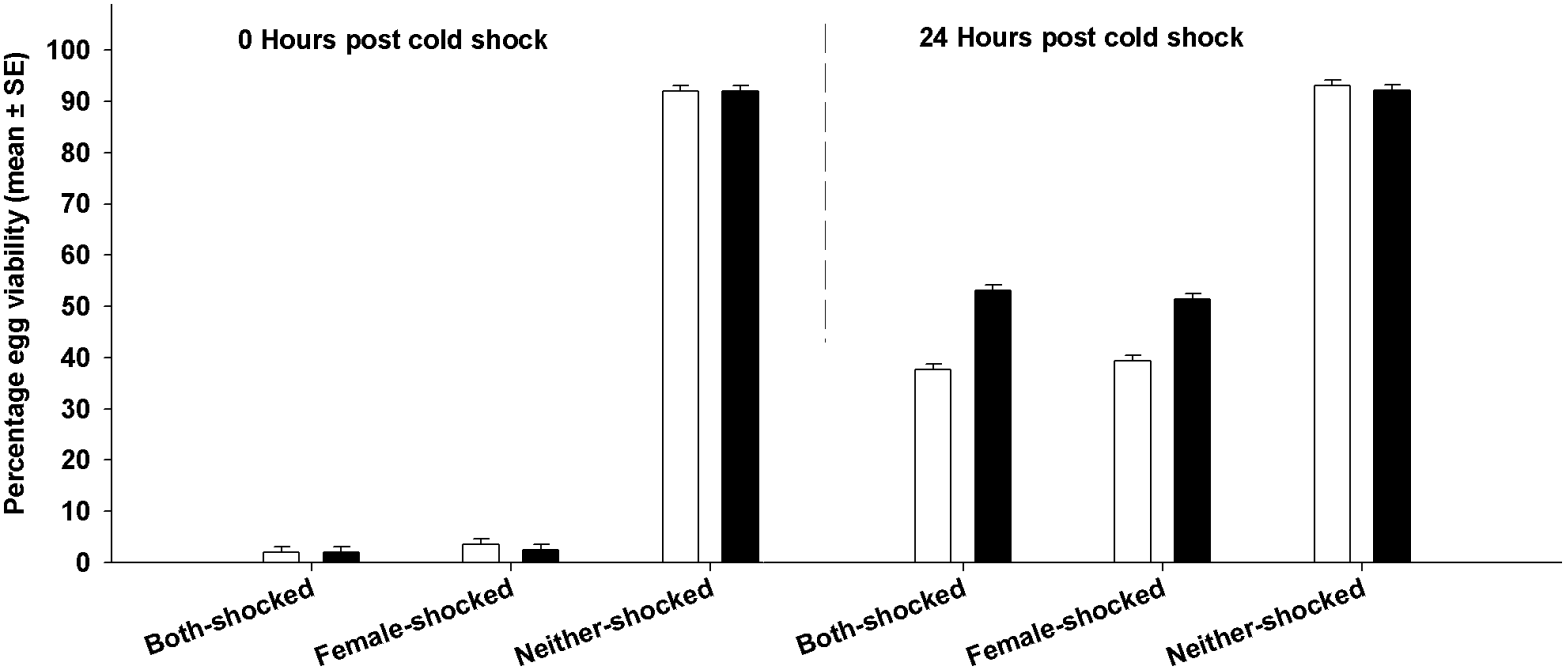
Effect of cold shock on fecundity. We assayed fecundity 0 and 24 hours post cold shock. Open bars represent FCB and black bars represent FSB populations. At 0 hours after cold shock, fecundity of females subjected to cold shock (Both-shocked and Female-shocked treatments) was high compared to that of females not subjected to cold shock (Neither-shocked treatment). However, there was no such difference 24 hours after cold shock. None of the differences between FCB and FSB populations were significant.

In our experiment, egg viability responded to selection. We found a significant effect of selection, treatment and period (Table 1). We also found a significant three way interaction between selection, treatment and period (Table 1). Eggs from FCB and FSB flies from Neither-shocked treatment had viability greater than 90% and there was no significant difference between them. Cold shock significantly reduced egg viability (Fig 1). At 0 hours post cold shock, the viability of eggs was extremely low (about 2%) and was not significantly different between FCB and FSB populations (Fig 1). At 24 hours post cold shock, the viability of the eggs had increased to about 45% (Fig 1). Additionally, 24 hours after cold shock, the viability of eggs from FSB populations was significantly higher than that of the FCB populations (Fig 1) indicating that the FSB populations had evolved a greater rate of recovery of egg viability.

**Table 1:** Effect of cold shock on egg viability (Experiment 1).

Effect	SS	MS Num	DF Num	DF Den	*F* ratio	*p*
Selection (Sel)	742.70	742.70	1	5.19	183.74	**< 0.01**
Treatment (Trt)	169973.46	84986.73	2	8.03	355.79	**< 0.01**
Block (Blk)	999.99	250.00	4	4.28	0.78	0.59
Period (Per)	33932.82	33932.82	1	4.02	145.34	**< 0.01**
Sel × Trt	494.49	247.22	2	8.30	9.07	**0.09**
Sel × Blk	14.49	3.62	4	4.31	0.12	0.97
Sel × Per	866.71	866.71	1	4.21	44.66	**<0.01**
Trt × Blk	1947.86	243.48	8	8.92	1.68	0.230
Trt × Per	16246.98	8123.49	2	8.06	61.84	**< 0.01**
Blk × Per	951.88	237.97	4	7.96	1.74	0.23
Sel × Trt × Blk	218.60	27.33	8	8	1.71	0.23
Sel × Trt × Per	528.91	264.46	2	8.51	16.41	**<0.01**
Sel × Blk × Per	77.27	19.32	4	8	1.21	0.38
Trt × Blk × Per	1069.31	133.66	8	8	8.38	**<0.01**
Sel × Trt × Blk × Per	127.63	15.95	8	108	0.68	0.71

Summary of results from a four-factor mixed model ANOVA on egg viability data using Selection (FCB and FSB), Treatment (Both-shocked, Female-shocked and Neither-shocked) and Period (0 and 24 hours post cold shock) as fixed factors crossed with random Blocks (1–5).

SS: Numerator sum of squares, MS Num: Numerator mean square, DF Num: Numerator degrees of freedom, DF Den: Denominator degrees of freedom.

The comparison between Both-shocked and Female-shocked treatments yields interesting insights. Twenty four hours post shock, egg viability was not significantly different between Both-shocked and Female-shocked treatments (Fig 1). It is important to note that the in Female-shocked treatment, the females interacted with healthy, non-shocked males, while in the Both-shocked treatment females interacted with males subjected to cold shock. Thus, our results indicate that recovery of egg viability is the same irrespective of whether the females interacted with healthy males or cold shocked males.

**Fig 2:**
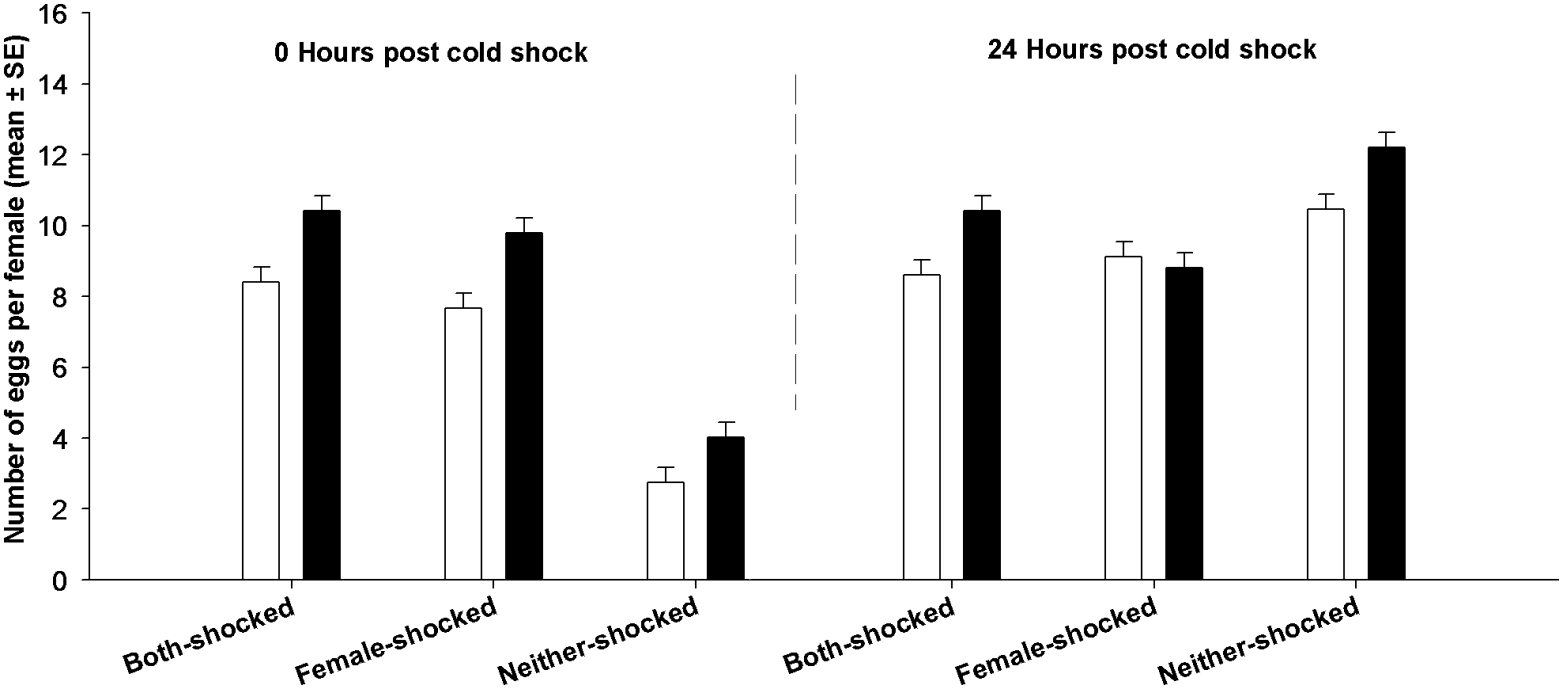
Effect of cold shock on egg viability. We assayed egg viability 0 and 24 hours post cold shock. Open bars represent FCB and black bars represent FSB populations. Viability of eggs from Neither-shocked treatment was high and not different between FCB and FSB populations. At 0 hours post cold shock, viability of eggs from the Both-shocked and Female-shocked treatment was very low and not different between FCB and FSB populations. By 24 hours post cold shock, egg viability improved and the FSB populations had significantly higher egg viability than the FCB populations.

In contrast to egg viability, fecundity remained unresponsive to selection. We found no significant effects of selection, block or period (Table 2, Fig 2). The effect of treatment was marginally significant (Table 2). However, we found a significant treatment **×** period interaction. Multiple comparisons using Tukey's HSD indicated that at 0 hours post cold shock, individuals subjected to cold shock had significantly higher fecundity compared to Neither-shocked individuals. However, 24 hours post cold shock, this difference in fecundity had disappeared (Fig 2). Females in the Female-Shocked treatment interacted with healthy males post cold shock while females from the Both-shocked treatment interacted with cold shocked males. However, fecundity of females from these two treatments (i.e. Female-shocked and Both-shocked) was not significantly different indicating that the type of male that the females interact with does not affect fecundity.

**Table 2:** Effect of cold shock on fecundity (Experiment 1).

Effect	SS	MS Num	DF Num	DF Den	*F* ratio	*p*
Selection (Sel)	85.19	85.19	1	4.03	1.97	0.23
Treatment (Trt)	127.29	63.64	2	8.17	4.51	**0.05**
Block (Blk)	92.11	23.03	4	5.63	0.13	0.97
Period (Per)	309.73	309.73	1	4.01	2.19	0.21
Sel × Trt	7.02	3.51	2	9.008	1.38	0.30
Sel × Blk	176.26	44.07	4	1.55	24.20	0.07
Sel × Per	5.25	5.25	1	4.72	2.90	0.15
Trt × Blk	114.00	14.25	8	7.88	0.69	0.69
Trt × Per	551.95	275.97	2	8.12	13.63	**<0.01**
Blk × Per	576.80	144.20	4	7.33	7.24	**0.01**
Sel × Trt × Blk	19.52	2.4	8	8	1.05	0.47
Sel × Trt × Per	15.75	7.87	2	9.05	3.26	0.09
Sel × Blk × Per	6.81	1.70	4	8	0.73	0.59
Trt × Blk × Per	164.32	20.54	8	8	8.84	**<0.01**
Sel × Trt × Blk × Per	18.58	2.32	8	108	0.34	0.95

Summary of results from a four-factor mixed model ANOVA on fecundity (eggs per female) data using Selection (FCB and FSB), Treatment (Both-shocked, Female-shocked and Neither-shocked) and Period (0 and 24 hours post cold shock) as fixed factors crossed with random Blocks (1–5).

**Fig 3:**
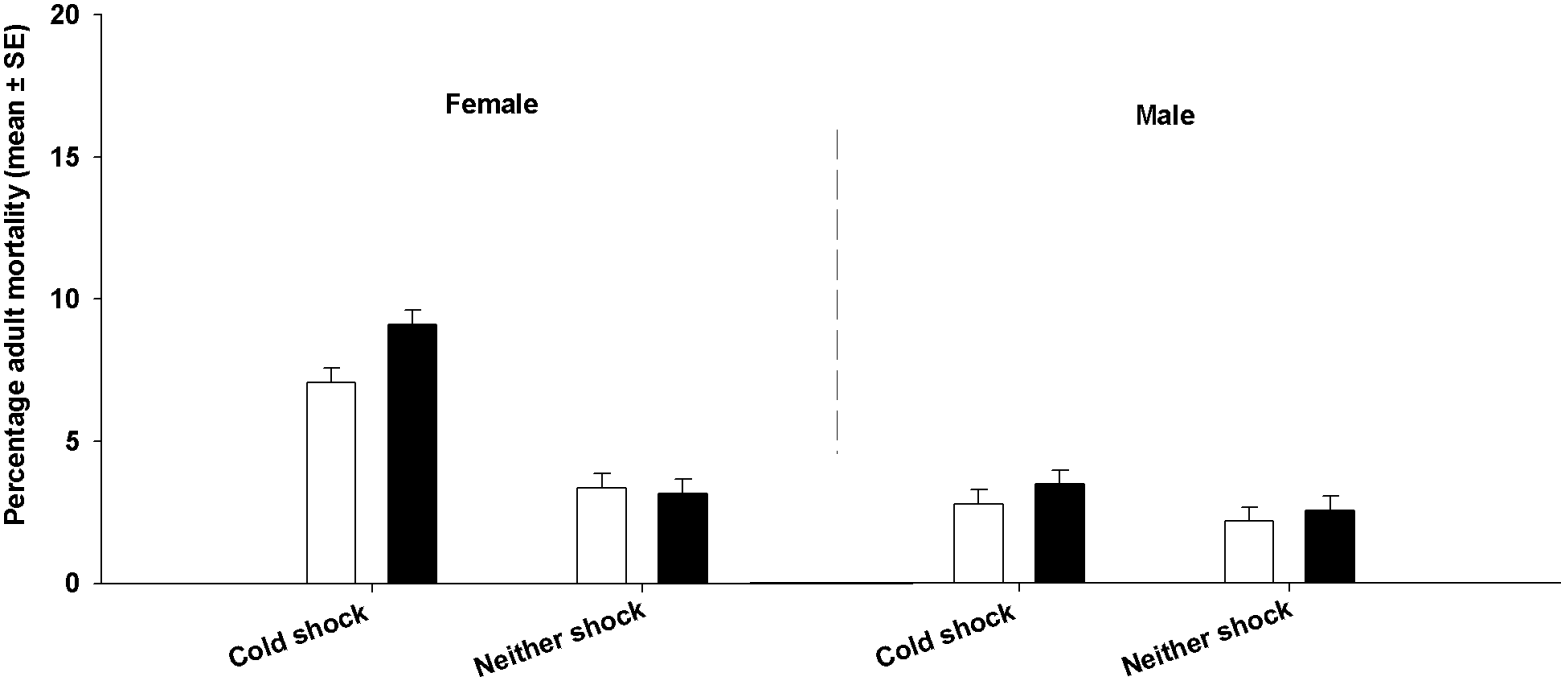
Effect of cold shock on adult mortality. Mortality of adults subjected to cold shock was significantly higher than the mortality of adults not subjected to cold shock. However, none of the differences between FCB (open bars) and FSB (closed bars) populations were significant.

To see if adult mortality post cold shock was different between the FSB and FCB populations, we used data from the Both-shocked and Neither-shocked treatments. We did not use the data from the Female-shocked treatment because in this treatment only females were shocked and combined with healthy males. Adult mortality did not differ significantly between FSB and FCB populations (Table 3, Fig 3). Treatment had a significant effect on mortality. We found that mortality of males and females that were not subjected to cold shock was low (about 2.5–3.5%) and cold shock increased the mortality of individuals. Mortality of females subjected to cold shock was about 7–9% while that of males was about 3–3.5%, leading to a significant Treatment by Sex interaction. Thus, while the overall mortality of adults due to cold shock was low, females died more often due to cold shock compared to males.

**Table 3:** Effect of cold shock on adult mortality (Experiment 1).

Source	SS	MS Num	DF Num	DF den	*F* ratio	*p*
Selection (Sel)	9.82	9.82	1	4.17	1.39	0.30
Treatment (Trt)	177.34	177.34	1	4.14	21.14	**0.01**
Block (Blk)	217.68	54.42	4	0.44	7.50	0.48
Sex	3.34	3.34	1	4.11	0.31	0.61
Sel × Trt	13.36	13.36	1	4.12	1.42	0.30
Sel × Blk	28.19	7.05	4	3.15	0.56	0.71
Sel × Sex	0.12	0.12	1	4.13	0.01	0.91
Trt × Blk	33.69	8.42	4	2.49	0.83	0.59
Trt × Sex	86.73	86.73	1	4.18	13.55	**0.02**
Blk × Sex	43.36	10.84	4	2.37	1.13	0.50
Sel × Trt × Blk	37.96	9.49	4	4	1.64	0.32
Sel × Trt × Sex	7.76	7.76	1	4.20	1.34	0.31
Sel × Blk × Sex	35.77	8.94	4	4	1.55	0.34
Trt × Blk × Sex	25.58	6.40	4	4	1.11	0.46
Sel × Trt × Blk × Sex	23.10	5.77	4	72	0.85	0.50

Summary of results from a four-factor mixed model ANOVA on adult mortality data using Selection (FCB and FSB), Treatment (Both-shocked and Neither-shocked) and Sex (male and female) as fixed factors crossed with random Blocks (1–5).

We found significant effect of Selection regime and Treatment on the number of mating pairs observed. Flies subjected to cold shock showed nearly twice as many mating pairs as flies not subjected to cold shock (mean ± SE; Both-shocked = 65.66 ± 2.84; Neither-shocked = 32.83 ± 2.84 (Table 4). Under both treatments, the FSB populations showed higher number of mating pairs compared to FCB populations (mean ± SE; Both-shocked treatment: FCB = 62.53 ± 2.53; FSB = 68.8 ± 2.53; Neither-shocked treatment: FCB = 29.46 ± 2.53; FSB = 36.2 ± 2.53). However, the selection × treatment interaction was not significant (Table 4). Thus, our results indicate that the mating frequency of the FSB populations has evolved to be higher even under Neither-shocked conditions.

**Table 4:** Effect of cold shock on number of mating pairs observed (Experiment 1).

Effect	SS	MS Num	DF Num	DF Den	*F* ratio	*p*
Selection (Sel)	576.14	576.14	1	4.48	21.24	**<0.01**
Treatment (Trt)	14700.38	14700.38	1	4.06	66.76	**<0.01**
Block (Blk)	12743.76	3185.94	4	1.79	19.70	0.06
Sel × Trt	0.74	0.74	1	4.14	0.01	0.93
Sel × Blk	104.76	26.19	4	4	0.30	0.87
Trt × Blk	893.57	223.39	4	4	2.54	0.19
Sel × Trt × Blk	351.42	87.86	4	36	1.24	0.31

Summary of results from a three-factor mixed model ANOVA on the number of mating pairs observed using Selection (FCB and FSB) and Treatment (Both-shocked and Neither-shocked) as fixed factors crossed with random Blocks (1–5).

### Experiment 2: Larval survivorship post cold shock has not evolved

We wanted to test whether cold shock can also affect larva to adult survivorship. Unlike egg viability, larval survivorship was not affected by treatment (Table 5. Survivorship of larvae from the two treatments was high (>90%). Additionally, there was no significant difference between FCB and FSB populations in larval survivorship (mean percentage ± SE; Cold shock treatment: FCB = 96.400 ± 0.642; FSB = 95.800 ± 0.642; Control treatment: FCB = 93.333 ± 0.642 FSB = 95.667 ± 0.642).

**Table 5:** Effect of cold shock on larval survivorship (Experiment 2).

Effect	SS	MS Num	DF Num	DF Den	*F* ratio	*p*
Selection (Sel)	37.56	37.56	1	4	2.45	0.19
Treatment (Trt)	128.00	128	1	4	3.58	0.13
Block (Blk)	1270.89	317.72	4	1.92	10.42	0.10
Sel × Trt	107.56	107.56	1	4	5.22	0.08
Sel × Blk	61.33	15.33	4	4	0.74	0.61
Trt × Blk	143.11	35.78	4	4	1.74	0.30
Sel × Trt × Blk	82.44	20.61	4	180	1.01	0.40

Summary of results from a three-factor mixed model ANOVA on larval survivorship data using Selection (FCB and FSB) and Treatment (Cold shock and Control) as fixed factors crossed with random Blocks (1–5).

### Experiment 3: Selected females are not better at protecting eggs/stored sperm from cold shock

**Fig 4:**
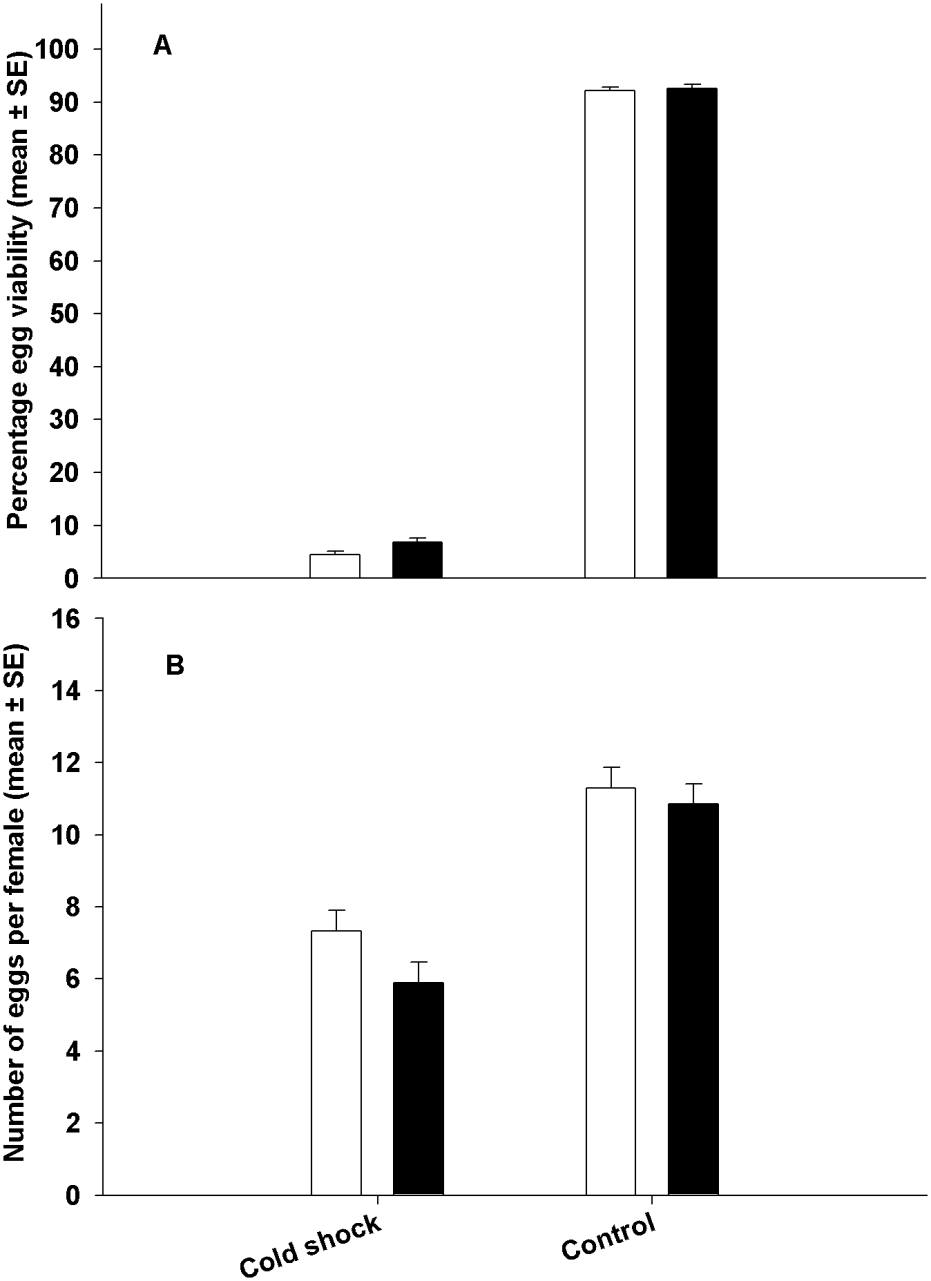
Effect of cold shock on egg viability and fecundity. To quantify egg viability (A) and fecundity (B) Females were held without access to males post cold shock. In this experiment, females were allowed to interact with males initially. However, after cold shock (or control treatment), females were not allowed to interact with males. We then assayed their egg viability (A) and fecundity (B) 24 hours later. Egg viability and fecundity of females subjected to cold shock and not allowed to interact with males thereafter was significantly lower. We found no significant differences in egg viability or fecundity between FCB (open bars) and FSB (closed bars) in either of the treatments. These results are in contrast to the results presented in Figs 1 and 2 wherein females had access to males post cold shock. Thus, taken together, these results indicate that interaction with males post cold shock is necessary for egg viability and fecundity to improve.

In this experiment we allowed females to interact with males until the time they were subjected to cold shock (or a control treatment at 25°C). However, post cold shock (or 25°C treatment), we denied the females access to males. We assayed egg viability and fecundity of these females 24 hours post cold shock. We found no significant effect of selection on egg viability or fecundity of the FSB and FCB regimes (Table 6). However, treatment had a significant effect on both egg viability and fecundity. When assayed without cold shock, egg viability and fecundity were high (Fig 4). However, when females were subjected to cold shock, there was a drastic decline in both egg viability and fecundity (Table 6, Fig 4). We found no significant Selection × Treatment interaction, indicating that the decline in egg viability and fecundity (due to cold shock treatment) was similar in the FCB and FSB populations (Table 6, Fig 4). Thus, when females were denied access to mates post cold shock, egg viability and fecundity remained extremely low even 24 hours post cold shock in both FCB and FSB populations. This clearly indicates that mating post cold shock is necessary to restore egg viability and fecundity.

**Table 6:** Egg viability and fecundity post cold shock (Experiment 3).

Trait	Effect	SS	MS Num	DF Num	DF Den	*F* ratio	*p*
(A)	Selection (Sel)	29.57	29.57	1	4	5.85	0.07
Egg	Treatment (Trt)	112781.61	112781.61	1	4	8465.06	**< 0.01**
viability	Block (Blk)	6.91	1.73	4	1.62	0.17	0.94
	Sel × Trt	15.78	15.78	1	4	1.97	0.23
	Sel × Blk	20.23	5.06	4	4	0.63	0.67
	Trt × Blk	53.29	13.32	4	4	1.67	0.32
	Sel × Trt × Blk	31.99	8.00	4	40	1.50	0.22
(B)	Selection (Sel)	13.59	13.59	1	4	4.50	0.10
Fecundity	Treatment (Trt)	298.79	298.79	1	4	23.92	**<0.01**
	Block (Blk)	231.65	57.91	4	2.39	5.45	0.13
	Sel × Trt	3.69	3.69	1	4	0.75	0.43
	Sel × Blk	12.10	3.02	4	4	0.62	0.67
	Trt × Blk	49.98	12.49	4	4	2.55	0.19
	Sel × Trt × Blk	19.59	4.90	4	40	1.37	0.26

Summary of results from three-factor mixed model ANOVA on (A) Egg viability and (B) Fecundity (number of eggs per female). In this experiment, females were held without access to males post cold shock. We modeled Selection (FCB and FSB) and Treatment (Cold shock and Control) as fixed factors crossed with random Blocks (1–5).

### Experiment 4: Post cold shock, selected males are better at mating non-virgin females and siring progeny

The non-virgin females in this experiment came from a base population with a recessive eye color marker (scarlet). The females were previously mated to males from their own population. Thus any female that produced progeny with dominant eye color marker (red) would have mated with FCB or FSB males at least once. We found that the proportion of females that produced red eyed progeny was significantly more when the females were held with FSB males than when they were held with FCB males (mean ± SE; FSB = 0.219 ± 0.022; FCB = 0.065 ± 0.022; paired t test, t = 4.846, df = 4, *p* = 0.008).

Thus, after being subjected to cold shock, males from FSB populations were successful to mate with more non-virgin females compared to males from the FCB populations. Consequently the FSB males also sired greater proportion of progeny compared to the males from the FCB populations (mean ± SE; FSB = 0.1211 ± 0.011; FCB = 0.0382 ± 0.011; paired t test, t = 5.484, df = 4, *p* = 0.005).

## Discussion

Insect responses to cold stress have received considerable attention [12]. Recent studies have addressed the physiological, biochemical and genetic basis underlying the ability to survive cold stress among insects [25, 44–46]. In the present experimental evolution study we investigated the effect of cold shock on egg viability (percentage of eggs that hatch) and other important components of fitness- larval survivorship, adult mortality, fecundity and reproductive behavior. We used egg viability as an indicator of the ability of the flies to lay fertile eggs. We found that following a cold shock, egg viability and total number of matings were higher in the selected populations. The selected populations mated more even in the absence of a cold shock. The males from the selected populations were more successful in mating with non-virgin females and sired more progeny post cold shock. Adult mortality and fecundity post cold shock were unresponsive to selection.

In our study, during the normal maintenance regime, eggs were collected from the flies 24 hours post cold shock (see materials and methods section) to start the next generation. Therefore, the ability to lay fertile eggs 24 hours post cold shock is an important component of fitness under this selection regime and is affected by the ability of the flies to protect the gametes from cold shock and/or ability to produce gametes and successfully mate post cold shock. We found that egg viability of FSB populations increases at a faster rate over the first 24 hours post cold shock compared to FCB populations. One possible reason for the increased egg viability in the selected populations could be that the females of the selected populations are better at protecting their eggs/stored sperm from damage due to cold shock. For example, studies on *Drosophila pseudoobscura* [47] indicate that females in this species can store sperm through six months of cold weather. These sperm can then be used to fertilize eggs with the onset of warm weather. However this is unlikely to be the case in our populations. When FSB and FCB females are held without access to males post cold shock, viability of their eggs remains low (even 24 hours after cold shock) and is not significantly different from each other. Therefore, in our populations, improvement in egg viability happens only if the females have access to males post cold shock.

While males are necessary for egg viability to improve, the type of male that the females are held with (i.e., subjected to cold shock or non-shocked) does not matter. Egg viability is likely to be limited by the availability of high-quality (i.e., not damaged by cold shock) eggs and sperm. Given that FCB females held with shocked FCB male or non-shocked base line males have similar egg viability (and the FSB females held with shocked FSB male or non-shocked base line males have similar egg viability), it follows that availability of high-quality sperm is not likely a limiting factor. The upper limit in egg viability within FCB and FSB populations is set primarily by the females. However, the difference in egg viability between FCB and FSB populations could be due to differences in the abilities of males and/or females of the two regimes to recover from cold shock.

Since our results suggest that access to males (and thereby mating) is necessary for increase in egg viability, it follows that egg viability post cold shock in a population is likely to increase as (a) more females in the population mate and/or (b) females are able to produce more high quality eggs. In contrast to previous studies—which found that cold shock reduced mating frequency [14, 36], our results suggest that in *D*. *melanogaster*, when females are held with males post cold shock, the number of matings observed is significantly higher (compared to when non-shocked individuals are held together). This is consistent with the hypothesis that mating post cold shock is necessary to produce fertile eggs and can hence affect male and female fitness. We also observed that post cold shock, FSB populations mated more often compared to FCB populations (see results section). Thus the observed differences in egg viability between FSB and FCB populations post cold shock are at least partly due to the differences in their mating behavior. FSB populations also had significantly higher mating even without cold shock, clearly indicating that the basal mating frequency has evolved in these populations.

One possible reason for the observed results could be that copulation duration is higher in FSB populations. If this is true, then, it is quite possible that the same mating pairs are scored repeatedly, thereby artificially increasing the number of mating pairs in FSB populations. However, in a separate experiment, we quantified copulation duration in FSB and FCB populations with and without cold stress (data not shown). We found that the mean copulation duration did not differ between the selection regimes and the value varied between 15 to 17 minutes across the populations. In the present study, we checked for the number of mating pairs once every 30 minutes. Therefore, it is unlikely that copulation duration can explain the increased number of mating pairs in the FSB populations. While multiple studies have looked at the effect of high and low temperature on mating behavior [48–53], very few studies have looked at the effect of cold shock on mating behavior. To the best of our knowledge, ours is the first study to document the evolution of mating frequency in response to cold shock.

Increased mating rate (post cold shock) in the selected populations could be due to increased receptivity of the females or increased ability of the males to successfully mate or both. Our results suggest that the selected males (post cold shock) mate more often with non-virgin females and sire a greater proportion of the progeny. In addition to differences in mating rate, the ability to sire progeny is likely to be affected by the ability of the males to transfer fertile sperm. Previous studies suggest that cold shock can reduce sperm content in males of some insects [36]. In particular, *D*. *melanogaster* males have been shown to have no motile sperm up to 24 hours after cold shock [38]. Therefore, it is possible that males from the FSB populations sperm have also evolved the ability to either (a) protect their sperm during cold shock and/or (b) produce fertile sperm at a faster rate after cold shock. However, at this point of time we are unable to distinguish between these two possibilities.

Unlike egg viability, larval mortality and adult mortality did not evolve in our populations. These results are not surprising given that the cold shock treatment we use does not affect larval mortality and induces very low levels of adult mortality. This is in contrast to other studies which show an effect of cold stress on larval and adult survivorship. Evolution of cold tolerance in larvae of various *Drosophila* species is well studied [54]. Studies show that maintaining flies at 18°C reduces the viability of their eggs compared to those of flies maintained at 25°C [26]. Embryos and larvae are known to be more sensitive to cold stress than pupae or adults [27]. Similarly, a cold shock of -5°C for one hour has been shown to reduce adult survivorship by over 60% [25]. When flies are maintained at 4°C, significant adult mortality is observed within 5 days while in flies maintained at 11°C, significant adult mortality occurs only after 10–12 weeks [27]. However, other studies find that the cold tolerance of adults and larvae from populations evolving at high (29°C) and low (16°C) temperatures are not different from each other [55].

We also found that fecundity was unresponsive to selection. Females subjected to cold shock laid more eggs immediately post-shock compared to females that were not subjected to cold shock. Given that viability of the eggs laid immediately post cold shock is extremely low, the increased fecundity immediately post-shock could represent the females’ effort to discard damaged eggs. The effect of cold shock on fecundity/progeny production has been reported by multiple studies. In a study similar to ours [25] a cold shock at -5°C for one hour severely reduced the progeny production by female *D*. *melanogaster* over the first eight hours after cold shock. Other studies have subjected flies to prolonged periods of low temperature instead of an acute cold shock. Subjecting flies to 18°C temperature for 1–3 days reduces their progeny production during treatment. However, fecundity returns to normal levels after flies are returned to 25°C [26]. In another study, an exposure of 9 days at 4°C rendered the flies unable to produce any progeny when returned to 25°C. In addition, when adult flies were stored for prolonged periods of time (1 to 12 weeks) in low temperatures (4–11°C) and then returned to 25°C, they suffered a severe reduction in progeny production over the first two days of recovery [27]. Unlike a single bout of cold shock, repeated exposure to cold stress in *D*. *melanogaster* leads to a survival and reproduction trade-off [28]. Flies subjected to multiple bouts of cold stress with periods of recovery in between show a greater survivorship compared to flies subjected to a single long bout of cold stress. The increased survivorship comes at the cost of decreased intrinsic growth rate (*r*). This study also found that a single 2-hour exposure does not reduce *r*. Thus the results about the effect of cold stress on fecundity have been variable. Similarly, evolution of fecundity in response to selection for resistance to cold shock has also been variable across studies, with selected populations evolving higher fecundity in some (8) and lower fecundity in other (7) studies. Since these studies differ in many aspects including the base populations used, the details of the selection and the assay protocols, there could be multiple reasons for the variation in the results observed across these studies.

Our experiments clearly show that reproductive traits such as rate of recovery of egg viability, mating rate and male mating ability evolve in response to cold shock. However, the mechanisms underlying the responses are as yet not clear. More importantly, it is very likely that the improved rate of recovery of egg viability, mating rate and male mating ability of the FSB populations are likely to come at a cost. Such costs, if any, are yet to be explored.

## Conclusions

The present experimental evolution study looked at the evolution of important fitness components in response to selection for cold shock resistance. While larval survivorship, adult mortality and fecundity were unresponsive to selection, we found that egg viability and mating frequency evolved rapidly in the selected populations. Post cold shock, males from the selected populations had higher ability to mate and sire progeny compared to control males. Thus, clearly, reproductive behavior has evolved in our selected populations in response to selection for resistance to cold shock. Our results clearly illustrate the role of environmental stress in shaping the evolution of reproductive behavior of organisms.

## Supporting Information

S1 Fig Derivation of BRB, FSB and FCB populations. BRB Population was created by combining 100 males and 100 females from each of the 19 isofemale lines. After ten generations, the single BRB population was further split into five replicate populations called BRB1-5. After 35 generations of laboratory adaptation, we derived one FSB and one FCB population from each BRB population.
(PDF)

S2 Fig Maintenance regime of the FSB and FCB populations. The selected populations (FSB 1–5) are maintained on a 13 day discrete generation cycle. On 12^th^ day post egg collection flies are exposed to -5°C for one hour. After cold shock, flies are immediately transferred into cages provisioned with fresh food. Twenty four hours post cold shock, fresh food plate is provided in the cage for 18 hours, after which eggs are collected (at a density of 100 eggs per vial) to start the next generation. The maintenance of control populations (FCB 1–5) is identical to that of FSB populations except (a) on 12^th^ day post egg collection, control flies are exposed to 25°C for one hour (instead of -5°C for one hour) and (b) eggs are collected at a density of 70 eggs per vial.
(PDF)

S3 Fig Experimental design for the measurement of egg viability, fecundity and mating frequency post cold shock. On 12^th^ day post egg collection, both FSB (1–5) and FCB (1–5) flies were subjected to the treatments. Post treatment, flies were immediately transferred into cages and a fresh food plate was provided. This food plate was replaced with a new one six hours later. The eggs laid on the first food plate were counted and some of those eggs were used to estimate hatchability. Thus we obtained fecundity and egg hatchability values for the 0–6 hours post treatment period. A fresh food plate was provided between 24–30 hours post treatment to measure fecundity and egg hatchability as before. In the Both-Shocked and Neither-shocked treatment, we also noted the number of mating pairs in each cage, every 30 minutes, for 36 hours post treatment.
(PDF)
