# Supplementary figures and images for: Egg Viability, Mating Frequency and Male Mating Ability Evolve in Populations of Drosophila melanogaster Selected for Resistance to Cold Shock
Source: PLoS One. 2015 Jun 11;10(6):e0129992. doi: 10.1371/journal.pone.0129992 (PMC4466231; doi:10.1371/journal.pone.0129992)

**S1 Fig.**

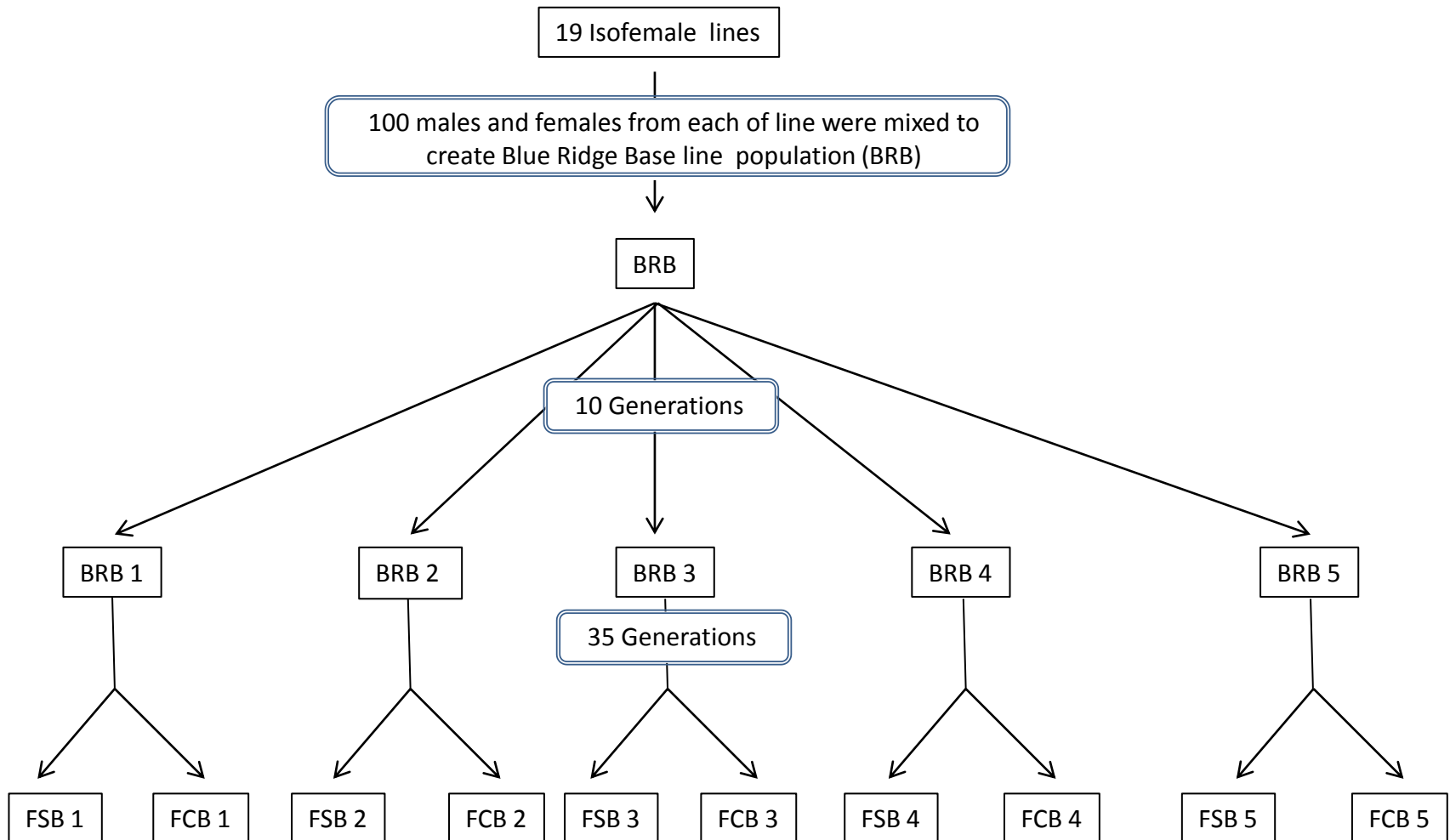

Supplement: S1 Fig — BRB Population was created by combining 100 males and 100 females from each of the 19 isofemale lines. After ten generations, the single BRB population was further split into five replicate populations called BRB1-5. After 35 generations of laboratory adaptation, we derived one FSB and one FCB population from each BRB population. (PDF) [file pone.0129992.s001.pdf]

S2 Fig.

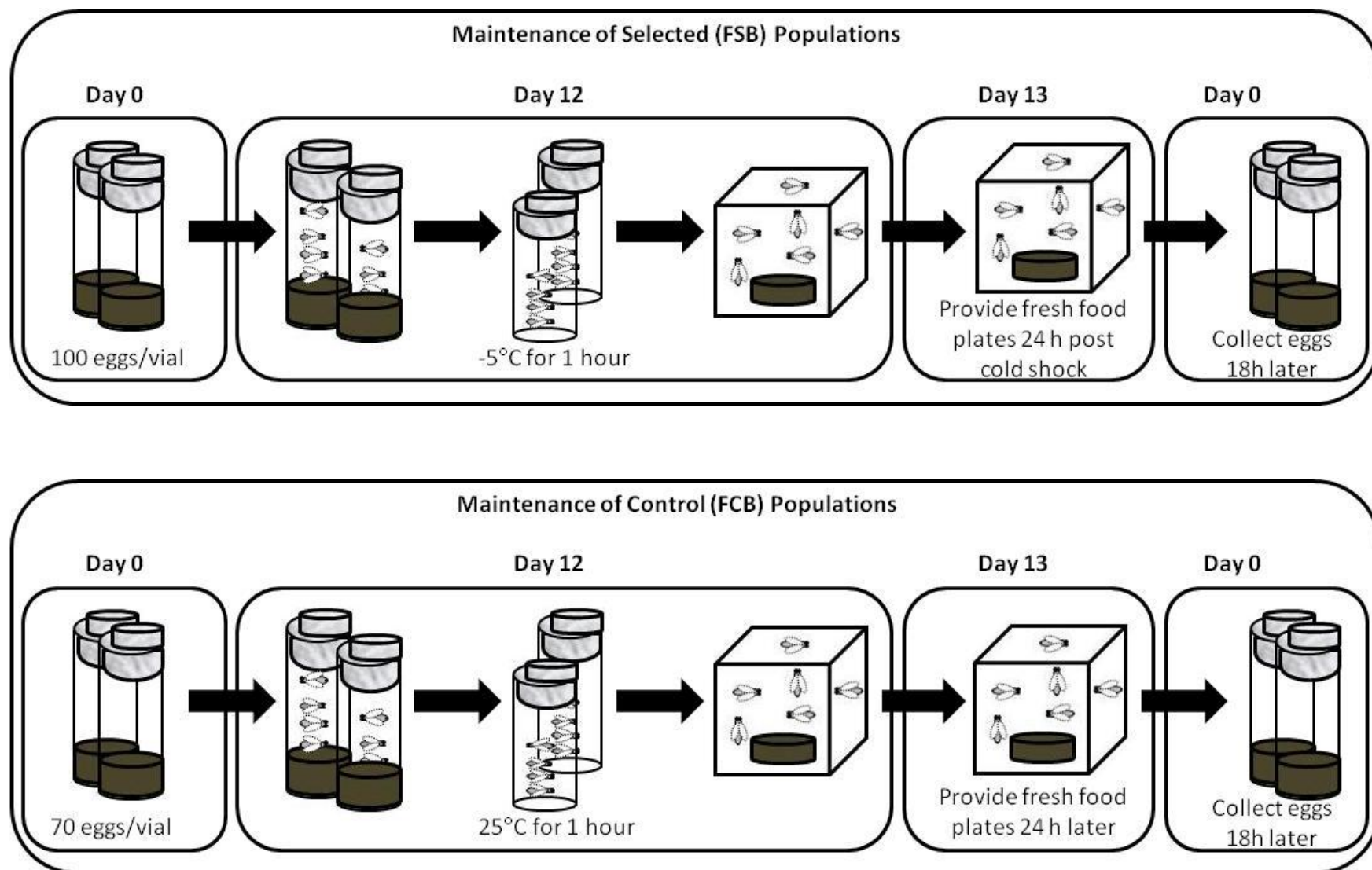

Supplement: S2 Fig — The selected populations (FSB 1–5) are maintained on a 13 day discrete generation cycle. On 12th day post egg collection flies are exposed to -5°C for one hour. After cold shock, flies are immediately transferred into cages provisioned with fresh food. Twenty four hours post cold shock, fresh food plate is provided in the cage for 18 hours, after which eggs are collected (at a density of 100 eggs per vial) to start the next generation. The maintenance of control populations (FCB 1–5) is identical to that of FSB populations except (a) on 12th day post egg collection, control flies are exposed to 25°C for one hour (instead of -5°C for one hour) and (b) eggs are collected at a density of 70 eggs per vial. (PDF) [file pone.0129992.s002.pdf]

S3 Fig.

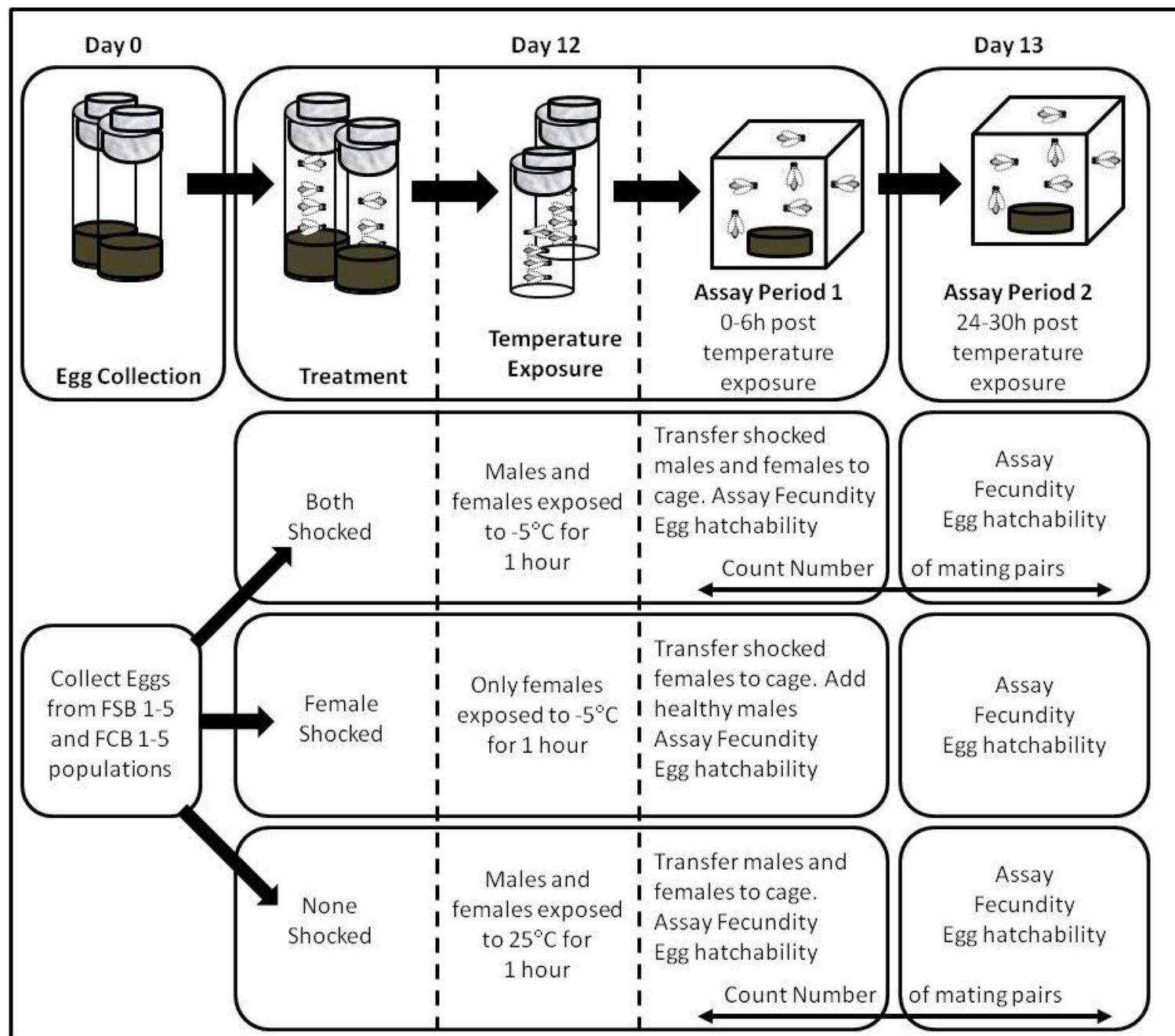

Supplement: S3 Fig — On 12th day post egg collection, both FSB (1–5) and FCB (1–5) flies were subjected to the treatments. Post treatment, flies were immediately transferred into cages and a fresh food plate was provided. This food plate was replaced with a new one six hours later. The eggs laid on the first food plate were counted and some of those eggs were used to estimate hatchability. Thus we obtained fecundity and egg hatchability values for the 0–6 hours post treatment period. A fresh food plate was provided between 24–30 hours post treatment to measure fecundity and egg hatchability as before. In the Both-Shocked and Neither-shocked treatment, we also noted the number of mating pairs in each cage, every 30 minutes, for 36 hours post treatment. (PDF) [file pone.0129992.s003.pdf]
